# Transcriptomic Analysis of Chicken Lungs Infected With Avian and Bovine *Pasteurella multocida* Serotype A

**DOI:** 10.3389/fvets.2020.00452

**Published:** 2020-08-11

**Authors:** Pan Li, Fang He, Chenlu Wu, Guangfu Zhao, Philip R. Hardwidge, Nengzhang Li, Yuanyi Peng

**Affiliations:** ^1^Chongqing Key Laboratory of Forage & Herbivore, College of Animal Science and Technology, Southwest University, Chongqing, China; ^2^The College of Life Sciences, Sichuan University, Chengdu, China; ^3^College of Veterinary Medicine, Kansas State University, Manhattan, KS, United States

**Keywords:** *Pasteurella multocida*, chicken lung, infection, transcriptome, immune response

## Abstract

*Pasteurella multocida* (*P. multocida*) is a common animal pathogen responsible for many animal diseases. Strains from different hosts exhibit disparate degrees of effect in other species. Here, we characterize an avian *P. multocida* serogroup A strain (PmQ) showing high lethality to chickens and a bovine *P. multocida* serogroup A strain (PmCQ2) with no lethality to chickens. We used RNA-seq to profile the transcriptomes of chicken lungs infected with PmQ and PmCQ2. A total of 1,649 differentially expressed genes (DEGs) due to PmQ infection (831 upregulated genes and 818 downregulated genes) and 1427 DEGs (633 upregulated genes and 794 downregulated genes) due to PmCQ2 infection were identified. Functional analysis of these DEGs demonstrated that the TNF signaling pathway, the toll-like receptor signaling pathway, complement and coagulation cascades, and cytokine–cytokine receptor interaction were both enriched in PmQ and PmCQ2 infection. STAT and apoptosis signaling pathways were uniquely enriched by PmQ infection, and the NOD-like receptor signaling pathway was enriched only by PmCQ2 infection. Cell-type enrichment analysis of the transcriptomes showed that immune cells, including macrophages and granulocytes, were enriched in both infection groups. Collectively, our study profiled the transcriptomic response of chicken lungs infected with *P. multocida* and provided valuable information to understand the chicken responses to *P. multocida* infection.

## Introduction

*Pasteurella multocida* (*P. multocida*) is a Gram-negative bacteria, first characterized by Louis Pasteur as the causative agent of fowl cholera in 1881 (1). *P. multocida* causes many animal diseases, such as fowl cholera, swine atrophic rhinitis, rabbit septicemia, and bovine pneumonia (2–4). Fowl cholera, caused by avian *P. multocida*, is a highly contagious disease of various domestic and wild bird species, resulting in great economic losses worldwide (5). In the case of peracute or acute infection, the lung lesions dominated by hemorrhages can be characteristic of fowl cholera (6). Antibiotics are still the main treatment for pasteurellosis due to the lack of effective multi-serotype vaccines (7). In some cases, *P. multocida* can cause human infection via animal bites and scratches (8, 9). Thus, further study on the mechanism of its pathogenesis is still needed.

*Pasteurella multocida* can be classified into five serotypes, A, B, D, E, and F, according to the specificity of capsular antigens (10). Different types of *P. multocida* strains often associated with different diseases depend on the infected host; for example, avian cholera is usually caused by *P. multocida* of serotype A and F, and swine atrophic rhinitis usually results from serotype D (2). Moreover, *P. multocida* isolated from certain hosts shows differing pathogenicity to other animals (11, 12). A *P. multocida* B:2 strain can kill cattle and buffaloes at a low dose but has no affect on chickens even at very high doses (13).

In a previous study, we isolated two *P. multocida* strains of serotype A, PmQ from tissues of a dead duck and PmCQ2 from pneumonic lungs of a beef cow (14, 15). Both strains showed high virulence to mice, but only PmQ was virulent in chickens and ducks. Here, we have used RNA-seq to investigate the chicken lung transcriptional response to these two *P. multocida* strains. This study provides comprehensive insights into the chicken immune response to *P. multocida* and demonstrates that different host-isolated strains show different virulence and may induce diverse immune responses.

## Materials and Methods

### Bacteria Strains and Culture Conditions

PmQ (GenBank accession No. CP033597) used in this study was isolated from tissues of a dead duck, and PmCQ2 (GenBank accession No. CP033599) was isolated from pneumonic lungs of a beef cow (16). Colonies were picked from Martin's broth agar plate, cultured overnight in Martin's broth aerobically at 37°C with shaking at 220 rpm, and counted by plating on Martin agar plates.

### Experimental Animals and Ethics Statement

A total of 150 commercial chickens (Ross 308, 7 days old), obtained from a commercial supplier (JinGang Village Farm, Chongqing), were used in our experiments. All animal experiments were carried out with the approval of the Animal Ethics and Research Committee of Southwest University (permit No. 11-1025), Chongqing, China.

### Chicken Challenge

Chickens were kept in wire cages separately in groups (*n* = 10 per group) with free access to food and water. Chickens of experimental groups were intraperitoneally infected with PmQ (PmQ-P) at a dosage of 1 × 10^3^ colony-forming units (CFU) in 200 μL saline and PmCQ2 (PmCQ2-P) at a dosage of 2 × 10^9^ CFU in 200 μL saline and then monitored for 7 days. The control group (P-C) was treated in parallel with sterile saline of equal volume.

Chickens infected with PmQ were sacrificed at 16 h post-infection (hpi) and lungs were collected. Chickens infected with PmCQ2 were sacrificed at 4, 8, 16, 24, and 48 hpi. Bacterial burdens were determined by homogenizing tissues and diluting ten-fold serially in saline. The different dilutions were plated in triplicate on Martin's broth agar and were incubated at 37°C for 24 h.

### Pathological Examinations

For histopathological examination, the lungs of chickens were collected and immediately fixed in 4% paraformaldehyde for 24 h, dehydrated in graded ethanol, and then embedded in paraffin wax. The tissues were sliced at 3 μm thickness and then stained with hematoxylin and eosin (H&E).

### RNA Isolation, Library Construction, and Illumina Deep Sequencing

About 50–100 mg of each lung were homogenized in liquid nitrogen. Total RNA was extracted using Trizol reagent (Invitrogen Life Technologies, USA) following the manufacturer's protocol. RNA integrity was confirmed by agarose gel electrophoresis and quantified by NanoDrop (NanoDrop 2000, Thermo Scientific). Following that, the Ribo-Zero rRNA Removal Kit (Illumina, San Diego, CA, USA) was used to remove rRNA. Then, 1 μg of each RNA sample was used to construct a cDNA library according to the manufacturer's recommendations. The cDNA library size was analyzed by using an Agilent 2100 Bioanalyzer and determining the effective concentration using qPCR (StepOnePlus Real-Time PCR Systems, Thermo Scientific). Finally, samples were sequenced on the Illumina sequencing platform (HiSeq 4000), and 150 bp paired-end reads were generated.

### Reads Filtering and Mapping

Read adaptors were removed by Cutadapt (Version 1.2.1), and reads with an average base quality lower than Q20 were discarded. Reads were mapped to the genome reference GCF_000002315.4, Gallus_gallus-5.0 from the Ensembl database (ftp://ftp.ebi.ac.uk/pub/databases/ena/wgs/public/aa/AADN05.fasta.gz). A genome index used Bowtie2, and then the clean reads were mapped to the genome by using Tophat2 (http://tophat.cbcb.umd.edu/). Mapping between reads and genomes that are within two mismatches were regarded as successful.

### Qualification and Identification of Differently Expression Genes (DEGs)

HTSeq 0.6.1p2 (http://www-huber.embl.de/users/anders/HTSeq) were used to obtain the read count of genes. To eliminate the effects of gene length and sample differences, reads per kilo bases per million (RPKM) was used to qualify the gene expression. Differential expression analysis was performed using estimateSizeFactors in DESeq (version 1.18.0) R package, and *p*-value and fold-change value were identified using the nbinomTest in DESeq with the R package. *P* < 0.05 and |log_2_foldchange| >1 were set as the threshold for DEG identification. Hierarchical cluster analysis was used to identify DEGs with certain patterns of expression under three different challenge groups using Heatmap based on R.

### Functional Annotation of DEGs

All DEGs were mapped to the gene ontology (GO) and KEGG ontology (KO) databases to obtain respective GO terms and KO classification and then enrichment analysis of the DEGs was performed using R based on hypergeometric distribution in the context of the entire reference genome. Gene clusters were subject to network analysis by using STRING (version 10.0) (17). Target genes were mapped to the STRING database to gain the relationship between each other and then drawn by Cytoscape (www.cytoscape.org).

To determine lung cell type enrichment, the transcript identifiers of the DEGs for each of the three major groups were converted into their associated gene names using BioMart software (http://uswest.ensembl.org/biomart/martview/). The three DEG lists were then input into the CTen database (18) to obtain the enriched cell types based on a highly expressed, cell-specific gene database. One-sided Fisher's exact test was used for enrichment analysis, and the CTen enrichment score was returned from the –log_10_ of the Benjamini-Hochberg (BH) adjusted *P*-values. The CTen database is originally based on human and mouse data and has previously been used for chickens (19, 20).

All sequence data discussed in this study have been deposited to NCBI's Sequence Read Archive (SRA) database, and the BioProject number is PRJNA597560.

### Quantitative Real-Time PCR (qRT-PCR)

Total RNA of the lungs was extracted, and then cDNA was synthesized using FastKing gDNA Dispelling RT SuperMix (Tiangen, Beijing, China). Thirteen immune response–related genes, including *Il6, Il1*β*, Il22, Il4i1, S100A9, Socs1, Socs3, AVD, AvBD1, AvBD2, AvBD4, TLR2A*, and *TLR4*, were selected to confirm RNAseq results. Criteria for gene selection were based on immune response function and significance in the RNAseq study. Beta-actin was used as a reference gene to normalize transcript levels of the target gene. Specific primers were designed according to the reference sequences in NCBI with Primer-BLAST under the criteria: (a) PCR product size 70–200 bp, (b) Melting temperatures 60 ± 2°C, and (c) primer must span an exon–exon junction (21). Primer sequences are listed in Table 1. The relative expression levels of genes are shown as a ratio of the target gene to the reference gene using the formula 2^−(ΔΔCt)^ (22).

**Table 1:** Primers used for validation of RNA-seq data.

**Gene name**	**Primer sequence (5′-3′)**	**Amplicon size (bp)**	**Accession number**
Il-6	F: GAAATCCCTCCTCGCCAAT	107	NM_204628.1
	R: CCCTCACGGTCTTCTCCATAA		
Il1β	F: TGGGCATCAAGGGCTACA	244	NM_204524.1
	R: TCGGGTTGGTTGGTGATG		
Il22	F: TCCAATGCCCATCAAGCC	84	NM_001199614.1
	R: TTCAGCCAAGGTGTAGGTGC		
Il4i1	F: ATAGGGTTGGTGGGAGGAT	177	NM_001099351.3
	R: GGATGCCGTTCACGAAGTA		
Il8l1	F: CAAGGCTGTAGCTGCTGTCA	95	NM_205018.1
	R: GGCATCGGAGTTCAATCG		
S100A9	F: GCTTTGGTGAAGTGATGCTCC	121	NM_001305151.1
	R: CAGTGGTTGTGCTGATGTTGG		
SOCS1	F: CCCATGAGAAGCTGAAGTCTG	137	NM_001137648.1
	R: ACGCCCCGTCTGAAAGTT		
SOCS3	F: CACCCCAAACGCACCTACTA	118	NM_204600.1
	R: CGTTGACAGTCTTACGGCAGA		
AVD	F: GCTCGCTGACTGGGAAAT	193	NM_205320.1
	R: ACGGTGAAGCCAAAGGTG		
AvBD1	F: TACCTGCTCCTCCCCTTCAT	104	NM_204993.1
	R: GCACAGAAGCCACTCTTTCG		
AvBD2	F: AGGCTTCTCCAGGGTTGTCT	133	NM_001201399.1
	R: CATTTGCAGCAGGAACGG		
AvBD4	F: ATGCTTACCTGGGGCTATGC	136	NM_001001610.2
	R: GACCGGTACAATGGTTCCCC		
TLR2A	F: ATCCTGGTGGTCGTTGGGTA	129	NM_204278.1
	R: GGAGACAAAAGCGTCGTAGC		
TLR4	F: CATCCCAACCCAACCACA	116	NM_001030693.1
	R: CTGAGCAGCACCAATGAGTAG		
β-actin	F: CAGACATCAGGGTGTGATGG	183	NM_205518.1
	R: TCAGGGGCTACTCTCAGCTC		

### Statistical Analysis

All data are displayed as means ± standard error of mean (SEM). GraphPad Prism 6.0 software was applied for all statistical analysis. Survival rates of chicks were evaluated using Kaplan-Meier analysis. All the other data were evaluated using unpaired, two-tailed Student's *t*-test. Significant differences were considered at *P* < 0.05. ^*^P < 0.05, ^**^P < 0.01, and ^***^P < 0.001.

## Results

### Mortality and Histopathology of Infected Chickens

**Figure 1:**
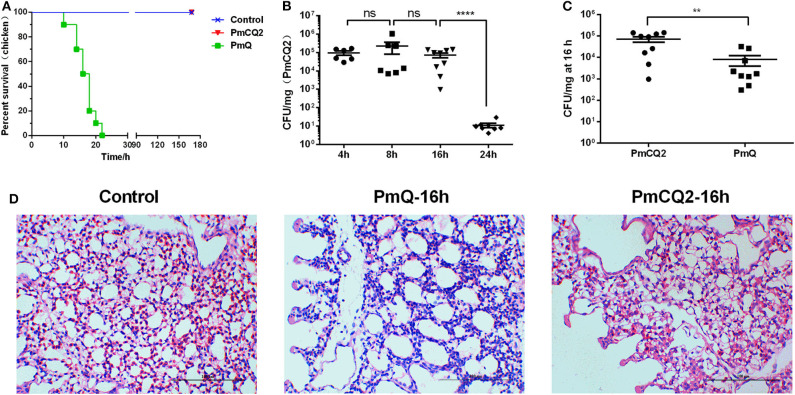
Chicken model infected with PmCQ2 and PmQ. **(A)** Survival curve of chickens challenged intraperitoneally with PmQ (1 × 10^3^ CFU), PmCQ2 (2 × 10^9^ CFU), and control (saline); **(B)** Bacterial burden of chicken lungs infected with PmCQ2 at 4, 8, 16, and 24 h; **(C)** Bacterial burden comparison of chicken lungs infected PmQ and PmCQ2 at 16 h; **(D)** The pulmonary histopathological examination of chicken at 16 h, stained with H&E (magnification 400X with a bar of 100 μm labeled). **p < 0.001, ****p < 0.00001, and ns, no significance.

Chickens infected with PmCQ2 and saline all survived, and PmQ-infected chickens all died rapidly within 24 h (Figure 1A). The bacterial burden of PmCQ2 in chicken lungs was approximately 10^5^ CFU/mg at 4 and 8 hpi, maintained a high density at 16 hpi, then decreased to 10^1^ CFU/mg at 24 hpi (Figure 1B). The bacterial load of PmQ in chicken lungs was about 10^4^ CFU/mg, on average, at 16 hpi (Figure 1C). Although chickens infected with PmQ had many inflammatory cell infiltrates, PmCQ2 infected chickens had few inflammatory cells detected (Figure 1D). PmCQ2-infected chickens all recovered with 7 days with no clinical signs of infection.

### Evaluation of Transcriptomic Sequencing Quality

A total of nine samples were generated from three biological replicates under three treatments; 44,374,891, 95,465,238, and 99,426,423 average clean reads were obtained from control and two infection group samples, respectively. The percentages mapped to the reference genome were more than 80% in all samples. A total of 19,119 genes were annotated in the Ensembl database for chicken genome. The average number of detected genes for individuals are 14,363, accounting for more than 75% of the reference genes. The percentage of uniquely mapped reads in all these samples was above 96%. Sequence data statistics are shown in Table 2.

**Table 2:** Statistic of RNA-seq output.

**Samples**	**Raw reads**	**Clean reads**	**Mapped reads**	**% of reads mapped**	**Detected genes**	**Transcriptome coverage %**
P-C1	43978738	43427176	35327533	81.35	13585	71.05
P-C2	43963256	43314098	34767932	80.27	13611	71.19
P-C3	47020662	46383400	37778449	81.45	13743	71.88
PmQ-P1	86425498	85407966	69118565	80.93	14390	75.27
PmQ-P2	104116160	102858060	83789800	81.46	14738	77.09
PmQ-P3	99436064	98129690	79668939	81.19	14784	77.33
PmCQ2-P1	98783656	97391824	78981365	81.1	14766	77.23
PmCQ2-P2	103872060	102309280	82633457	80.77	14593	76.33
PmCQ2-P3	100120672	98578166	79062279	80.2	15055	78.74

### Identification of DEGs in the Lungs After *P. multocida* Challenge

**Figure 2:**
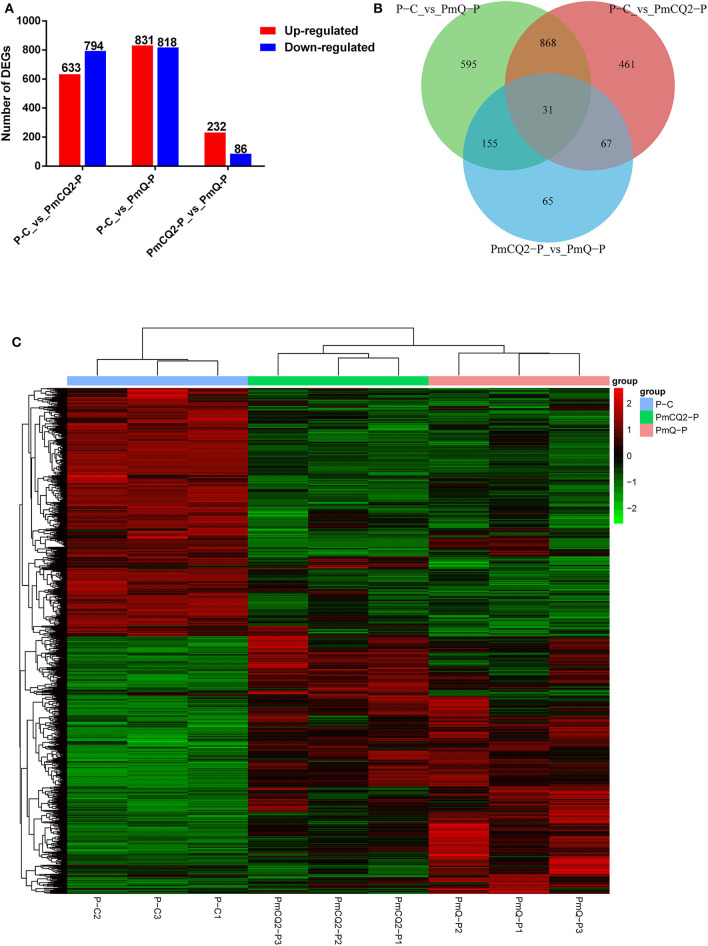
Differentially expressed genes (DEGs) results. **(A)** Numbers of up-/downregulated DEGs in three contrasts (P-C vs. PmQ-P, P-C vs. PmCQ2-P, and PmCQ2-P vs. PmQ-P). **(B)** Venn diagram of three contrasts. **(C)** The clustering of DEGs in a heat map.

Based on the criteria: |log_2_foldchange| >1 and *P* < 0.05, there were 1,649 DEGs in P-C vs. PmQ-P, of which 831 were upregulated and 818 were downregulated; 1,427 DEGs (633 upregulated genes and 794 downregulated genes) in P-C vs. PmCQ2-P; and 318 DEGs with 232 upregulated genes and 86 downregulated genes in PmCQ2-P vs. PmQ-P (Figure 2A). The description of total DEGs is displayed in Supplementary Table 1. A total of 899 DEGs were common in P-C vs. PmQ-P and P-C vs. PmCQ2-P (Figure 2B), of which 31 DEGs were also found in PmQ infection vs. PmCQ2. The full gene list is displayed in Supplementary Table 2. Unsupervised hierarchical clustering analysis of global gene expression profiles revealed that each of the three biological triplicates clustered together (Figure 2C).

### Experimental Validation of DEGs

To validate the results of RNA-seq, 13 immune response–related genes, including *Il6, Il1*β*, Il22, Il4i1, S100A9, Socs1, Socs3, AVD, AvBD1, AvBD2, AvBD4, TLR2A*, and *TLR4*, were chosen for qPCR detection. The gene expression patterns detected by qPCR were similar to those obtained from the RNA-seq results (Table 3).

**Table 3:** qRT-PCR validation.

**Gene**	**Contrast**	**qRT-PCR**	**RNA-seq**
Il6	P-C_vs_PmQ-P	89.22^*^	184.23^*^
	P-C_vs_PmCQ2-P	n.s.	n.s.
Il1β	P-C_vs_PmQ-P	12.98 (*p* = 0.2866)	113.09 (*p* = 0.0642)
	P-C_vs_PmCQ2-P	11.55^**^	8.68^***^
Il22	P-C_vs_PmQ-P	52.60^**^	17.73^*^
	P-C_vs_PmCQ2-P	n.s.	n.s.
Il4i1	P-C_vs_PmQ-P	372.52^**^	104.5^***^
	P-C_vs_PmCQ2-P	170.51 (*p* = 0.1051)	90.19^**^
S100A9	P-C_vs_PmQ-P	112.35^***^	35.18^***^
	P-C_vs_PmCQ2-P	74.92^**^	34.84^***^
SOCS1	P-C_vs_PmQ-P	10.83^**^	12.73^**^
	P-C_vs_PmCQ2-P	3.33^**^	n.s.
SOCS3	P-C_vs_PmQ-P	16.88^***^	20.61^**^
	P-C_vs_PmCQ2-P	7.90^*^	7.67^***^
AVD	P-C_vs_PmQ-P	79.42^*^	49.04^***^
	P-C_vs_PmCQ2-P	69.81^**^	58.53^***^
AvBD1	P-C_vs_PmQ-P	96.17^*^	30.01^***^
	P-C_vs_PmCQ2-P	24.00 (p=0.531)	28.18^***^
AvBD2	P-C_vs_PmQ-P	51.18^*^	16.58^***^
	P-C_vs_PmCQ2-P	11.05^***^	14.60^***^
AvBD4	P-C_vs_PmQ-P	6.98^**^	17.46^***^
	P-C_vs_PmCQ2-P	3.82^*^	18.42^***^
TLR2A	P-C_vs_PmQ-P	1.95^**^	1.93^**^
	P-C_vs_PmCQ2-P	5.95^**^	3.82^***^
TLR4	P-C_vs_PmQ-P	2.59^*^	4.16^***^
	P-C_vs_PmCQ2-P	10.98^*^	3.49^**^

*Fold-change between contrasts presented in third and fourth column. (P-C, N = 3; PmCQ2-P and PmQ-P, N = 3–6 per group)*.

* P < 0.05;

** P < 0.01; and

*** *P < 0.001*.

### Functional Analysis of DEGs

GO enrichment revealed that there were 34 GO terms enriched (of 3,864 annotated) in P-C vs. PmQ-P; 21 GO terms enriched (of 3,448 annotated) in P-C vs. PmCQ2-P (Table 4). The detailed information is listed in Supplementary Table 3. In both P-C vs. PmQ-P and P-C vs. PmCQ2-P, “defense response” and “immune system process” categories were predominant. Although categories including “inflammatory response,” “response to cytokine,” and “response to chemical” are only enriched in P-C vs. PmQ-P, the enriched GO terms in P-C vs. PmCQ2-P are more often associated with leukocytes and granulocytes, such as “leukocyte migration,” “granulocyte chemotaxis,” “leukocyte chemotaxis,” and “granulocyte migration.”

**Table 4:** Enriched GO terms analysis.

	**GO_Term**	**Corrected *P*-value**
P-C_vs_PmQ-P	GO:0006952 defense response	1.17E-14
	GO:0002376 immune system process	3.61E-11
	GO:0006955 immune response	9.46E-09
	GO:0009607 response to biotic stimulus	5.31E-08
	GO:0051707 response to other organism	0.000000271
	GO:0043207 response to external biotic stimulus	0.000000271
	GO:0009605 response to external stimulus	0.000000321
	GO:0006954 inflammatory response	0.0000024
	GO:0002682 regulation of immune system process	0.0000151
	GO:0050896 response to stimulus	0.000025
	GO:0009617 response to bacterium	0.0000285
	GO:0034097 response to cytokine	0.0000449
	GO:0042742 defense response to bacterium	0.0000463
	GO:0098542 defense response to other organism	0.0000904
	GO:0045087 innate immune response	0.00042
	GO:0042221 response to chemical	0.0006
	GO:0032101 regulation of response to external stimulus	0.00065
	GO:0070887 cellular response to chemical stimulus	0.00258
	GO:0071345 cellular response to cytokine stimulus	0.0039
	GO:0005615 extracellular space	0.00425
	GO:0034341 response to interferon-gamma	0.00432
	GO:0004867 serine-type endopeptidase inhibitor activity	0.00873
	GO:0044700 single organism signaling	0.00962
	GO:0023052 signaling	0.01086
	GO:0019221 cytokine-mediated signaling pathway	0.01126
	GO:0042555 MCM complex	0.01197
	GO:0002684 positive regulation of immune system process	0.01242
	GO:0007154 cell communication	0.01262
	GO:0010033 response to organic substance	0.01459
	GO:1903034 regulation of response to wounding	0.03252
	GO:0002252 immune effector process	0.03429
	GO:0033993 response to lipid	0.03495
	GO:0071310 cellular response to organic substance	0.0359
	GO:0031226 intrinsic component of plasma membrane	0.03944
P-C_vs_PmCQ2-P	GO:0006952 defense response	1.97E-08
	GO:0050900 leukocyte migration	0.00000936
	GO:0042742 defense response to bacterium	0.0000131
	GO:0002376 immune system process	0.0000155
	GO:0009617 response to bacterium	0.0003
	GO:0098542 defense response to other organism	0.00063
	GO:0006954 inflammatory response	0.00092
	GO:0071621 granulocyte chemotaxis	0.00094
	GO:0009605 response to external stimulus	0.0011
	GO:0051707 response to other organism	0.00123
	GO:0043207 response to external biotic stimulus	0.00123
	GO:0006955 immune response	0.00135
	GO:0030595 leukocyte chemotaxis	0.00136
	GO:0009607 response to biotic stimulus	0.0017
	GO:0006935 chemotaxis	0.00243
	GO:0042330 taxis	0.00279
	GO:0097530 granulocyte migration	0.00402
	GO:0060326 cell chemotaxis	0.00699
	GO:0005615 extracellular space	0.00773
	GO:0050829 defense response to Gram-negative bacterium	0.01376
PmCQ2–P_vs_PmQ–P	GO:0034341 response to interferon-gamma	0.00246
	GO:0060333 interferon-gamma-mediated signaling pathway	0.00612
	GO:0072562 blood microparticle	0.00706
	GO:0071346 cellular response to interferon-gamma	0.01742
	GO:0006952 defense response	0.0221
	GO:0051707 response to other organism	0.03572
	GO:0043207 response to external biotic stimulus	0.03572

**Figure 3:**
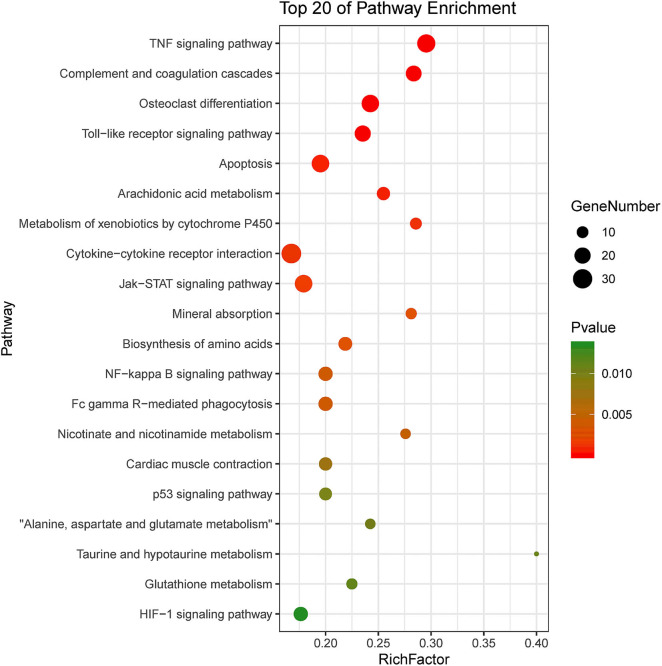
Top 20 enriched KEGG pathway in contrasts, P-C vs. PmQ-P. The numbers of DEGs in each pathway are counted, and rich factors and *P*-values are displayed.

**Figure 4:**
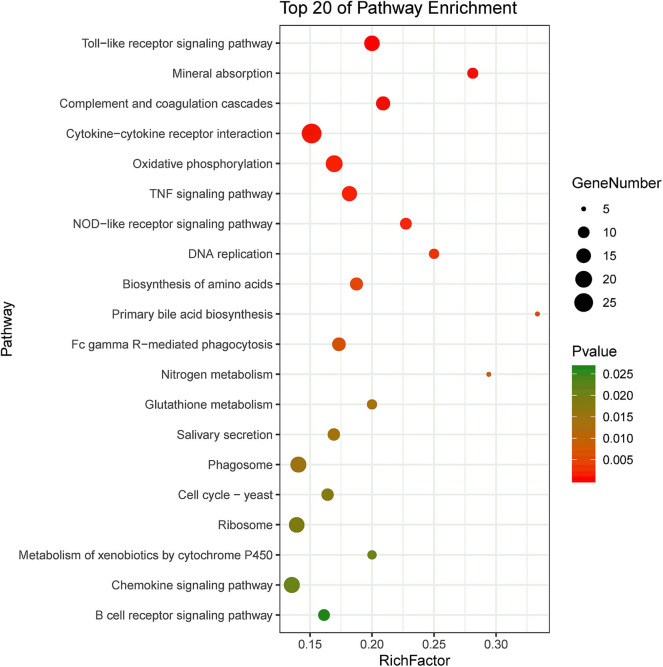
Top 20 enriched KEGG pathway in contrasts, P-C vs. PmCQ2-P. The numbers of DEGs in each pathway are counted, and rich factors and *P*-values are displayed.

KEGG pathway enrichment showed that 33 pathways were enriched in P-C vs. PmQ-P, of which the top 20 enriched pathways are displayed (Figure 3). Similarly, 30 pathways were enriched in P-C vs. PmCQ2-P (Figure 4). Many of these pathways are related to the immune system; signal transduction; the digestive system; and metabolism of lipids, amino acids, and xenobiotics. Both of the two contrasts clustered several conserved pathways, including “TNF signaling pathway,” “Toll-like receptor signaling pathway,” “Complement and coagulation cascades,” “Cytokine–cytokine receptor interaction,” “NF-kappa B signaling pathway,” “Fc gamma R-mediated phagocytosis,” and “Chemokine signaling pathway,” indicating that these pathways are related to *P. multocida* infection (Supplementary Table 4).

**Figure 5:**
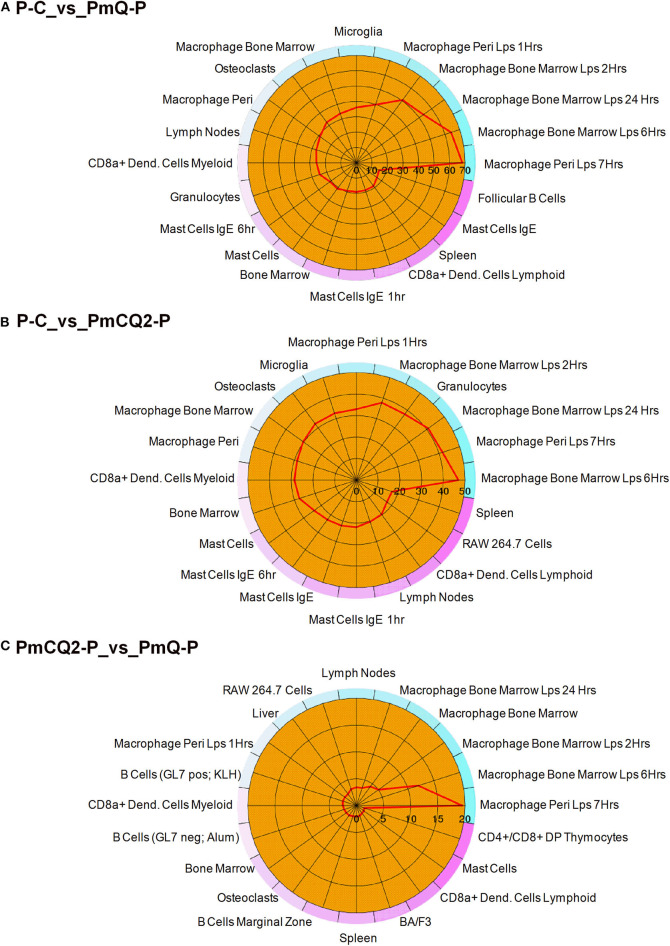
The 20 most enriched cell types. The enrichment scores (labeled inside the circle) is displayed in the –log_10_ of the Benjamini-Hochberg (BH) adjusted *P*-values. Cell/tissue types (labeled outside the circle) with an enrichment score (red line) greater than two were considered significant. **(A)** P-C vs. PmQ-P. **(B)** P-C vs. PmCQ2-P. **(C)** PmCQ2-P vs. PmQ-P.

**Figure 6:**
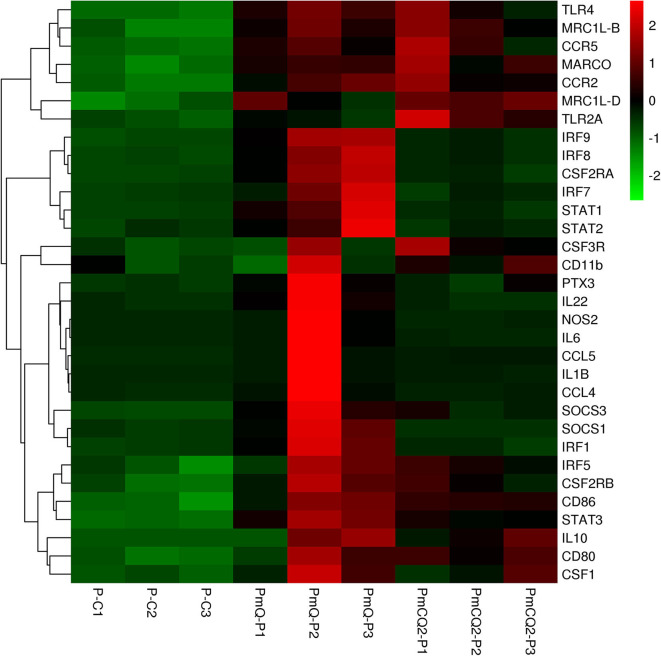
The expression level of macrophage-related genes displayed with heat map. The expression level (RPKM) of each gene were picked from Supplementary Table 7 and clustered by *z*-score.

Furthermore, cell type enrichment analysis based on upregulated DEGs showed that different types of macrophages are the most enriched immune cell type in both PmQ and PmCQ2 infected chicken vs. uninfected chicken (Figures 5A,B). Other immune related cell types, such as microglia, osteoclasts, granulocytes, lymph nodes, CD8a+ Dend. cells myeloid, bone marrow, and mast cells, were all significantly enriched (Supplementary Table 3). Specifically, comparing the PmCQ2 infected group with PmQ infection, the most enriched cell type was also focused on macrophages (Figure 5C), which suggests that macrophage may be the key immune cell upon *P. multocida* infection in chicken. Moreover, the expression level of macrophage related genes was displayed with a heat map (Figure 6). The clustered gene group of each gene showed that *TLR4, TLR2A, Macro, Ccr2, Ccr5, MRC1L-B*, and *MRC1L-D* were upregulated in both PmCQ2 and PmQ infected groups. Moreover, genes such as *Ifr9, Ifr8, Ifr7, CSF2RA, STAT1*, and *STAT2* were expressed to a greater extent in the PmQ infection group than PmCQ2, and *Il22, Il6, Nos2, Ccl4, Ccl5, Ifr1, Socs1*, and *Socs3* were increased only in PmQ infected chickens rather than PmCQ2.

## Discussion

*Pasteurella multocida* is a very common animal pathogen and is classified into five serotypes (A, B, D, E, and F) depending on the capsular antigen (10). Among them, serotype A tends to have a wide host range (2, 3). However strains isolated from non-avian hosts generally do not infect poultry, and the underling mechanisms are poorly understood (23). An avian *P. multocida* of serotype A, PmQ, isolated from tissues of a dead duck shows high virulence to poultry as well as to mammals. A bovine strain of serotype A, PmCQ2, from pneumonic lungs of a beef cow can cause severe pneumonia in mice (14, 16) but is non-lethal to chickens. It has been suggested that some of the differences in host susceptibility to *P. multocida* infection may be due to differences in the host response expressed in the lung during the early phase of infection (24). In the present study, we infected chickens with PmCQ2 at an extremely high dosage (2 × 10^9^ CFU), via the abdominal cavity to artificially create a chicken “infection model.” However, the PmCQ2 is quickly eliminated by the hosts, and a dramatic drop between 16 and 24 hpi of the bacterial load occurs. Whereas, the PmQ infected chickens died rapidly within 24 hpi. Therefore, transcripts of the chicken lungs at 16 hpi were used for RNA-seq.

The innate immune system is the first line in host defense and protects the host from bacterial infection (25). Previously, we found TLRs and inflammatory cytokines such as IL17, IL6, TNF-α, IFN-γ were significantly upregulated in mouse lungs infected with PmCQ2 (14). Another study also reports that IL6, IL17, and IL22 expression levels were significantly increased in the lungs of chicken challenged with avian *P. multocida* strain X73, which succumbed to acute infection (26). Consistent with these studies, the transcriptome of chicken lungs infected with PmQ also demonstrate that cytokines, such as IL6 and IL22, were significantly upregulated in PmQ-P vs. P-C, which implies PmQ infection induces a strong inflammatory reaction in the lungs. IL6 is a pleiotropic cytokine and can be both pro- and anti-inflammatory depending on the context (27). Impaired IL6 function causes enhanced susceptibility of mice to infection of *Listeria monocytogenes* (28), but excessive and sustained production of IL6 can be life-threatening, including systemic inflammatory response syndrome (SIRS) and chronic inflammatory diseases (29). IL22 is a member of the IL10 family and targets epithelial cells to induce various innate immune responses, which is essential for host defense against pathogen invasion (30). It can not only protect the tissue integrity and maintain mucosal homeostasis, but also act as a pro-inflammatory cytokine capable of amplifying the inflammatory reaction, and overproduction of IL22 may result in tissue damage (31). IL22 can mediate heme-associated iron scavenging away from pathogens during systematic infection (32, 33).

In PmCQ2 infected chickens, the expression level of IL22 and IL6 were unaffected. Similarly, chickens challenged with capsular-deficient strain 775 with low virulence also fail to increase IL6 and IL22 expression (26). Further verification using q-PCR indicated that IL6 and IL22 expression levels were unchanged during PmCQ2 infection (Supplementary Figure 1), which implies that PmCQ2 causes a lower inflammatory response. *CCL4, CCL5*, and *CCL28* were upregulated in PmQ and PmCQ2 infected chickens, but the expression level was increased to a high level in PmQ-P. CCL4, also known as macrophage inflammatory protein-1β (MIP-1β), is a chemoattractant for natural killer cells, monocytes, and a variety of other immune cells (34); CCL5 is chemotactic for T cells, eosinophils, and basophils and plays an active role in recruiting leukocytes into inflammatory sites (35); and CCL28 shows both antimicrobial activity and functions as a chemoattractant for T and B lymphocytes and migration of eosinophils. Together, these chemokines are ascribed to more immune cell infiltration, such as lymphocytes and eosinophils, in the PmQ infected chicken lungs. Whereas, the expression level of *CCL19* and *CCL26* were only increased in PmQ-P vs. P-C. CCL19 binding to its receptor CCR7 attracts dendritic cells to lymph nodes (36), and CCL26, also called macrophage inflammatory protein 4-alpha (MIP-4-alpha), can drive eosinophils and basophil migration by binding to CCR3 (37). In sum, these data suggest a different immune response of the host infected with the two *P. multocida* strains.

**Figure 7:**
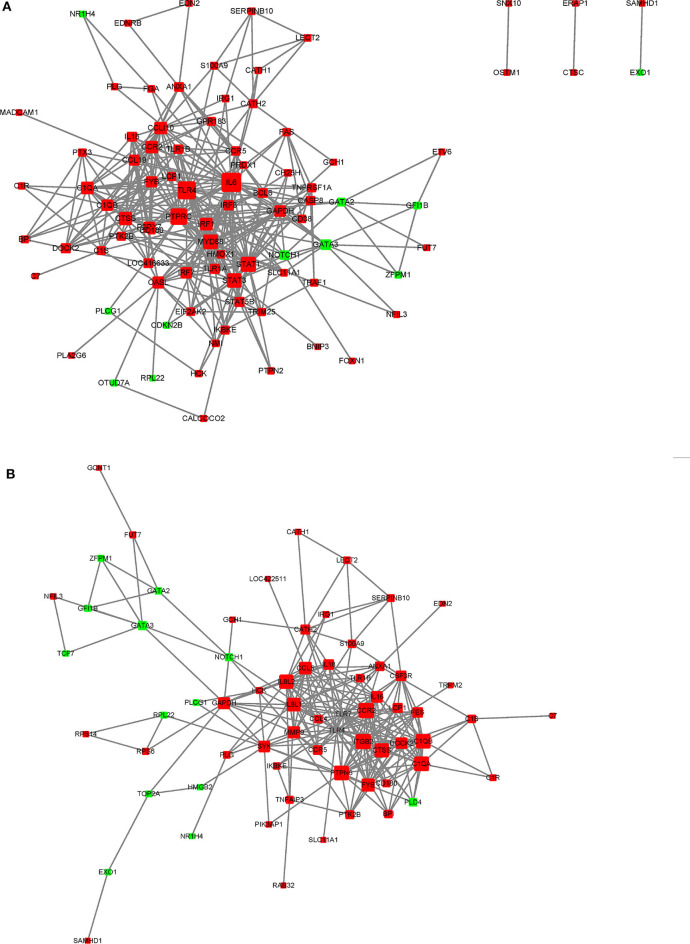
STRING network analysis on DEGs annotated in GO: immune system response. **(A)** P-C vs. PmQ-P. **(B)** P-C vs. PmCQ2-P. The nod represents proteins, bigger means more interaction with others. The red color means upregulated DEGs, and the green means downregulated DEGs.

In this study, a number of enrichment GO function categories were identified based on the DEGs of PmQ-P vs. P-C and PmCQ2-P vs. P-C. The protein–protein interactions based on DEGs annotated in immune system response of two infection groups (Figures 7A,B) indicated a different immune response interaction network. In PmQ infected chicken, IL6 and PTPRC (CD45) are in the center of the net. Upregulation of IL6 combined with STAT1 and STAT2/3, attributed to the key points of the Jak-STAT pathway, demonstrated that the STAT pathway was activated during *P. multocida* infection, which is consistent with our previous study (14). The activation of the STAT pathway can then trigger a signaling cascade required for immune response and inflammatory reaction (38), resulting in production of IFN-γ, contributing to bacterial clearance (39, 40). CD45, expressed on leukocytes, is a crucial player in the activation of T-cell receptor signaling by controlling the activation of the Src family protein-tyrosine kinases Lck and Fyn. CD45 deficiency can result in T- and B-lymphocyte dysfunction in the form of severe combined immune deficiency (41). Some immunomodulatory proteins, such as UL11 of human cytomegalovirus, can interact with CD45, resulting in function disruption of T-cells (42).

Different from *P. multocida* toxin (PMT) (43, 44), lipopolysaccharides isolated from *P. multocida* serotype A and B stains can induce apoptosis of bovine leukocytes, including lymphocytes, neutrophils, monocytes, and macrophages, resulting in tissue damage (45, 46). In the current study, genes (*FAS, APAF1, TNFRSF1A, CASP7, and CASP8*) related to apoptosis were significantly upregulated in PmQ infected chickens but not in chickens infected with PmCQ2. In contrast to our previous study, the apoptosis pathway is enriched in PmCQ2 infected mice with a deadly outcome (14). Together, these results hint that the PmQ infected chicken attempt to cause cell death of the host, leading to tissue lesions of the lungs.

The NOD-like receptors (NLRs) are a specialized group of intracellular innate immune receptors that mediate IL1β production against bacterial infection (47). In the present study, the NOD-like receptor signaling pathway was enriched alone in the PmCQ2 infected chicken, which is consistent with the previous study of PmCQ2 infected mice (14). *NLRC5*, which can act as a negative modulator of inflammatory pathways, is critical for LPS-induced IL-10 production in macrophages (48), which upregulated in PmCQ2 infection, which suggests it might contribute to the lower inflammatory reaction in PmCQ2 infection. Our previous study revealed the NLRP3 inflammasome of mouse peritoneal macrophages infected with PmCQ2 played a role in mediating IL1β against *P. multocida* infection (49). In line with the study, IL1β was also significantly increased in PmCQ2 infected chicken, which might responsible for PmCQ2 clearance.

Macrophages play a crucial role in sensing *P. multocida* and secretion of inflammatory factors (49). We found that L-serine supplementation can decrease the macrophage-mediated inflammatory reaction in PmCQ2 infected mice and reduce the lung damage (50). In the current study, cell type enrichment analysis based on DEGs showed that macrophages were the most significantly enriched cell types, suggesting that macrophages were also activated in chickens infected with two different host-isolated *P. multocida* strains (Figure 5). Moreover, when analyzing the DEGs between PmCQ2 and PmQ infected chicken, macrophage was still the most enriched cell type. However, the different outcomes of chicken challenged with PmCQ2 and PmQ hint that macrophage may play different roles. For example, NOS2, a key marker, and PTX3 and IRF5, two important regulators of M1 macrophage activation (51–53), were significantly overexpressed in PmQ infected chicken, tending to activate to more pro-inflammatory activation. Whereas, in the PmCQ2 infected chicken lung, M2 markers (MRC1LD, MRC1LB, and MACRO) and M1 markers (CD86 and CD80) were upregulated, implying a mixed activation of both M1 and M2 macrophage occurred. In Marek's disease virus (MDV) infection, macrophages derived from differentially sensitive chicken lines undergo different immune strategies, and the macrophages from resistant chicken lines activate different biological pathways and suppression of oncogenic potential (54). Macrophages can be activated into different states depending upon the microenvironment and stimulus encountered (55, 56), and the activation styles are often related to its morphology (57).

In addition to the immune response, metabolism was also affected upon *P. multocida* infection. For example, GAD1, a key enzyme that converts L-glutamate to GABA, used for succinic semialdehyde synthesis and eventually succinate, which is the main route of M1 macrophage and fundamental for stabilization of HIFα (58, 59), was significantly upregulated in PmQ infected chicken while GLUL (also known as glutamine synthetase, GS), highly expressed in M2 macrophages and important to M2 phenotype (60), is increased in PmCQ2 infected chicken. However, PmCQ2 infection also caused reduction of all NADH dehydrogenase subunits (ND1~ND6), implying the oxidative phosphorylation of chicken was impaired, which was unfavorable to M2 macrophage activation (61). A recent study reported that arachidonic acid can bind to myeloid differentiation factor-2 (MD2) and prevent MD2/TLR4 signaling activation, thus, it inhibits inflammatory response (62). Ten genes associated with arachidonic acid metabolism are upregulated in PmQ infected chickens, resulting in overproduction of a number of metabolites, for example, PGD2 (Prostaglandin D_2_), which can bind to its receptor DP2 and amplify inflammatory response in asthma (63). Overall, these results suggest macrophages might undergo different polarization and contribute to opposite outcomes of chickens.

In conclusion, different host-isolated serotype A *P. multocida* strains, bovine PmCQ2 and avian PmQ, exhibited opposite pathogenicity to birds. Additionally, this study also revealed several crucial pathways related to anti-infection and metabolisms were enriched and that macrophages might play a key role in *P. multocida* infection.

## Data Availability Statement

The datasets presented in this study can be found in online repositories. The names of the repository/repositories and accession number(s) can be found at: https://www.ncbi.nlm.nih.gov/, PRJNA597560.

## Ethics Statement

This animal study was reviewed and approved by Animal Ethics and Research Committee of Southwest University.

## Author Contributions

YP and NL conceived the experiments. PL conducted the experiments and wrote the paper. FH, CW, and GZ contributed to the experiments and data analysis. PH, YP, and NL supervised the project and revised the paper. All authors discussed the results and approved the final manuscript.

## Conflict of Interest

The sequencing service was provided by Personal Biotechnology Co., Ltd., Shanghai, China. The authors declare that the research was conducted in the absence of any commercial or financial relationships that could be construed as a potential conflict of interest.

## References

[1] Kubatzky KF. *Pasteurella multocida* and immune cells. Curr Top Microbiol Immunol. (2012) 361:53–72. 10.1007/82_2012_204 22427181

[2] Wilkie IW Harper M Boyce JD Adler B. *Pasteurella multocida*: diseases and pathogenesis. Curr Top Microbiol Immunol. (2012) 361:1–22. 10.1007/82_2012_216 22643916

[3] Wilson BA Ho M. *Pasteurella multocida*: from zoonosis to cellular microbiology. Clin Microbiol Rev. (2013) 26:631–55. 10.1128/CMR.00024-13 23824375 PMC3719492

[4] Peng Z Wang XR Zhou R Chen HC Wilson BA Wu B. *Pasteurella multocida*: Genotypes and Genomics. Microbiol Mol Biol Rev. (2019) 83:e00014–00019. 10.1128/MMBR.00014-19 31484691 PMC6759666

[5] Sellyei B Thuma A Volokhov D Varga Z. Comparative analysis of *Pasteurella multocida* isolates from acute and chronic fowl cholera cases in Hungary during the period 2005 through 2010. Avian Dis. (2017) 61:457–65. 10.1637/11674-051817-Reg.1 29337626

[6] Christensen JP Bisgaard M. Fowl cholera. Rev Sci Tech. (2000) 19:626–37. 10.20506/rst.19.2.1236 10935284

[7] Liu Q Hu Y Li P Kong Q. Identification of fur in *Pasteurella multocida* and the potential of its mutant as an attenuated live vaccine. Front Vet Sci. (2019) 6:5. 10.3389/fvets.2019.00005 30778390 PMC6369157

[8] Honnorat E Seng P Savini H Pinelli PO Simon F Stein A. Prosthetic joint infection caused by *Pasteurella multocida*: a case series and review of literature. BMC Infect Dis. (2016) 16:435. 10.1186/s12879-016-1763-0 27544345 PMC4992566

[9] Ryan JM Feder HM Jr. Dog licks baby. Baby gets *Pasteurella multocida* meningitis. Lancet. (2019) 393:e41. 10.1016/S0140-6736(19)30953-5 31226054

[10] Carter GR. Studies on *Pasteurella multocida*. I. A hemagglutination test for the identification of serological types. Am J Vet Res. (1955) 16:481–484. 13238757

[11] Okay S Kurt Kizildogan A. Comparative genome analysis of five *Pasteurella multocida* strains to decipher the diversification in pathogenicity and host specialization. Gene. (2015) 567:58–72. 10.1016/j.gene.2015.04.063 25917966

[12] Peng Z Liang W Wang F Xu Z Xie Z Lian Z. Genetic and phylogenetic characteristics of *Pasteurella multocida* isolates from different host species. Front Microbiol. (2018) 9:1408. 10.3389/fmicb.2018.01408 29997608 PMC6029419

[13] Aktories K Orth J Adler B Pasteurella multocida. Berlin; Heidelberg: Verlag; Springer (2012). p. 5

[14] Wu C Qin X Li P Pan T Ren W Li N. Transcriptomic analysis on responses of murine lungs to *Pasteurella multocida* infection. Front Cell Infect Microbiol. (2017) 7:251. 10.3389/fcimb.2017.00251 28676843 PMC5476747

[15] Du H Fang R Pan T Li T Li N He Q. Comparative genomics analysis of two different virulent bovine *Pasteurella multocida* isolates. Int J Genomics. (2016) 2016:4512493. 10.1155/2016/4512493 28070502 PMC5192330

[16] Li N Long Q Du H Zhang J Pan T Wu C. High and low-virulent bovine *Pasteurella multocida* capsular type A isolates exhibit different virulence gene expression patterns *in vitro* and *in vivo*. Vet Microbiol. (2016) 196:44–49. 10.1016/j.vetmic.2016.10.017 27939154

[17] Szklarczyk D Franceschini A Wyder S Forslund K Heller D Huerta-Cepas J. STRING v10: protein-protein interaction networks, integrated over the tree of life. Nucleic Acids Res. (2015) 43:D447–52. 10.1093/nar/gku1003 25352553 PMC4383874

[18] Shoemaker JE Lopes TJS Ghosh S Matsuoka Y Kawaoka Y Kitano H. CTen: a web-based platform for identifying enriched cell types from heterogeneous microarray data. BMC Genomics. (2012) 13:460. 10.1186/1471-2164-13-460 22953731 PMC3473317

[19] Deist MS Gallardo RA Bunn DA Dekkers JCM Zhou H Lamont SJ. Resistant and susceptible chicken lines show distinctive responses to Newcastle disease virus infection in the lung transcriptome. BMC Genomics. (2017) 18:989. 10.1186/s12864-017-4380-4 29281979 PMC5745900

[20] Zhang J Kaiser MG Deist MS Gallardo RA Bunn DA Kelly TR. Transcriptome analysis in spleen reveals differential regulation of response to Newcastle disease virus in two chicken lines. Sci Rep. (2018) 8:1278. 10.1038/s41598-018-19754-8 29352240 PMC5775430

[21] Ye J Coulouris G Zaretskaya I Cutcutache I Rozen S Madden TL. Primer-BLAST: a tool to design target-specific primers for polymerase chain reaction. BMC Bioinform. (2012 13:134. 10.1186/1471-2105-13-134 22708584 PMC3412702

[22] Lee SI Jang HJ Jeon MH Lee MO Kim JS Jeon IS. Transcriptional regulation of cathelicidin genes in chicken bone marrow cells. Poult Sci. (2016) 95:912–19. 10.3382/ps/pev361 26908883

[23] Ahir VB Roy A Jhala MK Bhanderi BB Mathakiya RA Bhatt VD. Genome sequence of *Pasteurella multocida* subsp *gallicida* Anand1_poultry. J Bacteriol. (2011) 193:5604–4. 10.1128/Jb.05706-11 21914901 PMC3187395

[24] Jens P Christensen Miki Bojesen A Bisgaard M Fowl Cholera. Philadelphia, PA: Elsevier (2008). p. 149–54.

[25] Mogensen TH. Pathogen recognition and inflammatory signaling in innate immune defenses. Clin Microbiol Rev. (2009) 22:240–73. 10.1128/CMR.00046-08 19366914 PMC2668232

[26] Petruzzi B Dalloul RA LeRoith T Evans NP Pierson FW Inzana TJ. Biofilm formation and avian immune response following experimental acute and chronic avian cholera due to *Pasteurella multocida*. Vet Microbiol. (2018) 222:114–23. 10.1016/j.vetmic.2018.07.005 30080666

[27] Hunter CA Jones SA. IL-6 as a keystone cytokine in health and disease. Nat Immunol. (2015) 16:448–57. 10.1038/ni.3153 25898198

[28] Hoge J Yan I Janner N Schumacher V Chalaris A Steinmetz OM. IL-6 controls the innate immune response against listeria monocytogenes via classical IL-6 signaling. J Immunol. (2013) 190:703–11. 10.4049/jimmunol.1201044 23241882

[29] Kang S Tanaka T Kishimoto T. Therapeutic uses of anti-interleukin-6 receptor antibody. Int Immunol. (2015) 27:21–9. 10.1093/intimm/dxu081 25142313

[30] Kim S Faris L Cox CM Sumners LH Jenkins MC Fetterer RH. Molecular characterization and immunological roles of avian IL-22 and its soluble receptor IL-22 binding protein. Cytokine. (2012) 60:815–27. 10.1016/j.cyto.2012.08.005 22980486

[31] Eidenschenk C Rutz S Liesenfeld O Ouyang W. Role of IL-22 in microbial host defense. Curr Top Microbiol Immunol. (2014) 380:213–36. 10.1007/978-3-662-43492-5_10 25004820

[32] Kugelberg E. Infection: IL-22 controls iron scavenging. Nat Rev Immunol. (2017) 17:146–7. 10.1038/nri.2017.15 28216614

[33] Sakamoto K Kim YG Hara H Kamada N Caballero-Flores G Tolosano E. IL-22 controls iron-dependent nutritional immunity against systemic bacterial infections. Sci Immunol. (2017) 2:eaai8371. 10.1126/sciimmunol.aai8371 28286877 PMC5345941

[34] Bystry RS Aluvihare V Welch KA Kallikourdis M Betz AG. B cells and professional APCs recruit regulatory T cells via CCL4. Nat Immunol. (2001) 2:1126–32. 10.1038/ni735 11702067

[35] Maghazachi AA AlAoukaty A Schall TJ. CC chemokines induce the generation of killer cells from CD56(+) cells. Eur J Immunol. (1996) 26:315–9. 10.1002/eji.1830260207 8617297

[36] Robbiani DF Finch RA Jager D Muller WA Sartorelli AC Randolph GJ. The leukotriene C(4) transporter MRP1 regulates CCL19 (MIP-3beta, ELC)-dependent mobilization of dendritic cells to lymph nodes. Cell. (2000) 103:757–68. 10.1016/s0092-8674(00)00179-3 11114332

[37] Kitaura M Suzuki N Imai T Takagi S Suzuki R Nakajima T. Molecular cloning of a novel human CC chemokine (eotaxin-3) that is a functional ligand of CC chemokine receptor 3. J Biol Chem. (1999) 274:27975–80. 10.1074/jbc.274.39.27975 10488147

[38] Kiu H Nicholson SE. Biology and significance of the JAK/STAT signalling pathways. Growth Factors. (2012) 30:88–106. 10.3109/08977194.2012.660936 22339650 PMC3762697

[39] Zeng X Gu H Peng LS Yang Y Wang N Shi Y. Transcriptome profiling of lung innate immune responses potentially associated with the pathogenesis of *Acinetobacter baumannii* acute lethal Pneumonia. Front Immunol. (2020) 11:708. 10.3389/fimmu.2020.00708 32391015 PMC7188829

[40] Hu X Ivashkiv LB. Cross-regulation of signaling pathways by interferon-gamma: implications for immune responses and autoimmune diseases. Immunity. (2009) 31:539–50. 10.1016/j.immuni.2009.09.002 19833085 PMC2774226

[41] Rheinlander A Schraven B Bommhardt U. CD45 in human physiology and clinical medicine. Immunol Lett. (2018) 196:22–32. 10.1016/j.imlet.2018.01.009 29366662

[42] Gabaev I Steinbruck L Pokoyski C Pich A Stanton RJ Schwinzer R. The human cytomegalovirus UL11 protein interacts with the receptor tyrosine phosphatase CD45, resulting in functional paralysis of T cells. PLoS Pathog. (2011) 7:e1002432. 10.1371/journal.ppat.1002432 22174689 PMC3234252

[43] Hildebrand D Heeg K Kubatzky KF. *Pasteurella multocida* toxin manipulates T cell differentiation. Front Microbiol. (2015) 6:1273. 10.3389/fmicb.2015.01273 26635744 PMC4652077

[44] Preuss I Hildebrand D Orth JH Aktories K Kubatzky KF. *Pasteurella multocida* toxin is a potent activator of anti-apoptotic signalling pathways. Cell Microbiol. (2010) 12:1174–185. 10.1111/j.1462-5822.2010.01462.x 20331638

[45] Periasamy S Praveena PE Singh N. Effects of *Pasteurella multocida* lipopolysaccharides on bovine leukocytes. Microb Pathog. (2018) 119:225–32. 10.1016/j.micpath.2018.04.030 29678740

[46] Praveena PE Periasamy S Kumar AA Singh N. Cytokine profiles, apoptosis and pathology of experimental *Pasteurella multocida* serotype A1 infection in mice. Res Vet Sci. (2010) 89:332–9. 10.1016/j.rvsc.2010.04.012 20447665

[47] Chen G Shaw MH Kim YG Nunez G. NOD-like receptors: role in innate immunity and inflammatory disease. Ann Rev Pathol Mech Dis. (2009) 4:365–98. 10.1146/annurev.pathol.4.110807.092239 18928408

[48] Benko S Magalhaes JG Philpott DJ Girardin SE. NLRC5 limits the activation of inflammatory pathways. J Immunol. (2010) 185:1681–91. 10.4049/jimmunol.0903900 20610642

[49] Fang R Du H Lei G Liu Y Feng S Ye C. NLRP3 inflammasome plays an important role in caspase-1 activation and IL-1beta secretion in macrophages infected with *Pasteurella multocida*. Vet Microbiol. (2019) 231:207–13. 10.1016/j.vetmic.2019.03.019 30955811

[50] He F Yin Z Wu C Xia Y Wu M Li P. l-serine lowers the inflammatory responses during *Pasteurella multocida* infection. Infect Immun. (2019) 87:e00677–19. 10.1128/IAI.00677-19 31570555 PMC6867830

[51] Murray PJ. Macrophage polarization. Annu Rev Physiol. (2017) 79:541–66. 10.1146/annurev-physiol-022516-034339 27813830

[52] Ferrer MF Thomas P Lopez Ortiz AO Errasti AE Charo N Romanowski V. Junin virus triggers macrophage activation and modulates polarization according to viral strain pathogenicity. Front Immunol. (2019) 10:2499. 10.3389/fimmu.2019.02499 31695702 PMC6817498

[53] Wei J Tang D Lu C Yang J Lu Y Wang Y. Irf5 deficiency in myeloid cells prevents necrotizing enterocolitis by inhibiting M1 macrophage polarization. Mucosal Immunol. (2019) 12:888–96. 10.1038/s41385-019-0169-x 31086271 PMC7746522

[54] Chakraborty P Kuo R Vervelde L Dutia BM Kaiser P Smith J. Macrophages from susceptible and resistant chicken lines have different transcriptomes following Marek's disease virus infection. Genes. (2019) 10:74. 10.3390/genes10020074 30678299 PMC6409778

[55] Arora S Dev K Agarwal B Das P Syed MA. Macrophages: their role, activation and polarization in pulmonary diseases. Immunobiology. (2018) 223:383–96. 10.1016/j.imbio.2017.11.001 29146235 PMC7114886

[56] Murray PJ Wynn TA. Protective and pathogenic functions of macrophage subsets. Nat Rev Immunol. (2011) 11:723–37. 10.1038/nri3073 21997792 PMC3422549

[57] McWhorter FY Wang TT Nguyen P Chung T Liu WF. Modulation of macrophage phenotype by cell shape. Proc Natl Acad Sci USA. (2013) 110:17253–58. 10.1073/pnas.1308887110 24101477 PMC3808615

[58] Tannahill GM Curtis AM Adamik J Palsson-McDermott EM McGettrick AF Goel G. Succinate is an inflammatory signal that induces IL-1 beta through HIF-1 alpha. Nature. (2013) 496:238. 10.1038/nature11986 23535595 PMC4031686

[59] Meiser J Kramer L Sapcariu SC Battello N Ghelfi J D'Herouel AF. Pro-inflammatory macrophages sustain pyruvate oxidation through pyruvate dehydrogenase for the synthesis of itaconate and to enable cytokine expression. J Biol Chem. (2016) 291:3932–46. 10.1074/jbc.M115.676817 26679997 PMC4759172

[60] Palmieri EM Menga A Martin-Perez R Quinto A Riera-Domingo C De Tullio G. Pharmacologic or genetic targeting of glutamine synthetase skews macrophages toward an M1-like phenotype and inhibits tumor metastasis. Cell Rep. (2017) 20:1654–66. 10.1016/j.celrep.2017.07.054 28813676 PMC5575233

[61] Viola A Munari F Sanchez-Rodriguez R Scolaro T Castegna A. The metabolic signature of macrophage responses. Front Immunol. (2019) 10:1462. 10.3389/fimmu.2019.01462 31333642 PMC6618143

[62] Zhang YL Chen HJ Zhang WX Cai Y Shan PR Wu D. Arachidonic acid inhibits inflammatory responses by binding to myeloid differentiation factor-2 (MD2) and preventing MD2/toll-like receptor 4 signaling activation. Biochim Biophys Acta Mol Basis Dis. (2020) 1866:165683. 10.1016/j.bbadis.2020.165683 31953218

[63] Brightling CE Brusselle G Altman P. The impact of the prostaglandin D2 receptor 2 and its downstream effects on the pathophysiology of asthma. Allergy. (2020) 75:761–8. 10.1111/all.14001 31355946

